# Selective Maternal Seeding and Rearing Environment From Birth to Weaning Shape the Developing Piglet Gut Microbiome

**DOI:** 10.3389/fmicb.2022.795101

**Published:** 2022-04-25

**Authors:** Wei Chen, Jingyun Ma, Yiming Jiang, Li Deng, Ning Lv, Jinming Gao, Jian Cheng, Juan Boo Liang, Yan Wang, Tian Lan, Xindi Liao, Jiandui Mi

**Affiliations:** ^1^College of Animal Science, National Engineering Research Center for Breeding Swine Industry, South China Agricultural University, Guangzhou, China; ^2^Guangdong Provincial Key Lab of Agro-Animal Genomics and Molecular Breeding, Guangzhou, Chain; ^3^Ministry of Agriculture Key Laboratory of Tropical Agricultural Environment, South China Agricultural University, Guangzhou, China; ^4^Institute of Virology, Helmholtz Zentrum München, German Research Center for Environmental Health, Neuherberg, Germany; ^5^Institute of Virology, Technical University of Munich, Munich, Germany; ^6^Laboratory of Sustainable Animal Production and Biodiversity, Institute of Tropical Agriculture and Food Security, University Putra Malaysia, Serdang, Malaysia; ^7^Key Laboratory of Animal Health Aquaculture and Environmental Control, Guangzhou, China

**Keywords:** piglet, gut microbiome, maternal seeding, rearing environment, colonization

## Abstract

The acquisition and development of the mammalian microbiome early in life are critical to establish a healthy host-microbiome symbiosis. Despite recent advances in understanding microbial sources in infants, the relative contribution of various microbial sources to the colonization of the gut microbiota in pigs remains unclear. Here, we longitudinally sampled the microbiota of 20 sow-piglet pairs (three piglets per sow) reared under identical conditions from multiple body sites and the surrounding weaning environment from birth to 28 days postpartum (1,119 samples in total). Source-tracking analysis revealed that the contribution of various microbial sources to the piglet gut microbiome gradually changed over time. The neonatal microbiota was initially sparsely populated, and the predominant contribution was from the maternal vaginal microbiota that increased gradually from 69.0% at day 0 to 89.3% at day 3 and dropped to 0.28% at day 28. As the piglets aged, the major microbial community patterns were most strongly associated with the sow feces and slatted floor, with contributions increasing from 0.52 and 9.6% at day 0 to 62.1 and 33.8% at day 28, respectively. The intestinal microbial diversity, composition, and function significantly changed as the piglets aged, and 30 age-discriminatory bacterial taxa were identified with distinctive time-dependent shifts in their relative abundance, which likely reflected the effect of the maternal and environmental microbial sources on the selection and adaptation of the piglet gut microbiota. Overall, these data demonstrate that the vaginal microbiota is the primary source of the gut microbiota in piglets within 3 days after birth and are gradually replaced by the sow fecal and slatted floor microbiota over time. These findings may offer novel strategies to promote the establishment of exogenous symbiotic microbes to improve piglet gut health.

## Introduction

The mammalian gastrointestinal tract is a diverse and complex ecosystem of microbes that play fundamental roles in metabolic, developmental, and physiological processes affecting host health (Clemente et al., 2012; Flint et al., 2012; Buffie and Pamer, 2013). The first 2–3 years of life is a critical time for the colonization of the gut microbiota (Cox et al., 2014; Gensollen et al., 2016; Torow and Hornef, 2017). In addition, microbial exposure early in life plays an important role in the transition to a stable adult gut microbiota (Connell and Slatyer, 1977; Biasucci et al., 2008). Aberrant neonatal gut microbiota has been reported to be linked to many diseases during childhood and later in life, including inflammatory bowel diseases (IBDs; Morgan et al., 2012; Gevers et al., 2014; Kolho et al., 2015). While the importance of gut microbiota colonization early in life to host health is not in question, the main sources of infant gut microbiota and the relative contribution of various microbial sources remain largely unexplored.

The establishment of the gut microbiota is a complex and dynamic process from a near sterile state early in life to a highly dense gut microbiota (Cilieborg et al., 2012; Tanya et al., 2012). The first major exposure of the neonate to microbes occurs during the birthing process and is highly dependent on delivery mode. The gut of vaginally delivered neonates is enriched with *Lactobacillus* and *Prevotella*, which resemble the maternal vaginal microbiota (Biasucci et al., 2010; Dominguez-Bello et al., 2010; Milani et al., 2017). In contrast, the gut of cesarean-section-born infants is instead colonized by microbes from the maternal skin or the hospital environment, such as staphylococci, streptococci, or propionibacteria (Dominguez-Bello et al., 2010; Bäckhed et al., 2015). In addition, bifidobacterial communities are present in both infant feces and maternal milk, indicative of a vertical transmission route from the maternal gastrointestinal tract (Ted et al., 2014; Milani et al., 2015). Thus, intimate contact with the mother, breastfeeding and general environmental exposure play critical roles in the early gut microbial acquisition in the infant.

The pig is both a major source of meat for human consumption and a valuable biomedical model. The gut microbiota plays essentially important roles in pig health, including providing energy for the host, producing metabolites through fermentation, and improving the capacity to resist pathogens (Kim and Isaacson, 2015; Yang et al., 2017). Early gut microbiota colonizers are crucial for the formation of a mature microbial community and thus affect the health and productivity of pigs (Guevarra et al., 2019). However, despite the emerging understanding of the gut microbiota of pigs, few studies have characterized the development of the early gut microbiota of piglets, and no current studies have systematically characterized the main sources of the piglet gut microbiota early in life.

Thus, a large-scale study was conducted to investigate the contribution of the multiple maternal and environmental sources to the colonization of the gut microbiota of piglets from birth to 4 weeks of age. Additionally, we assessed the dynamic changes in the composition and potential metabolic function of the piglet gut microbiota longitudinally. In this study, we collected a total of 1,119 samples from sows (vagina, feces, milk, and breast skin), the environment of the nursing house (air, water, and slatted floor), and piglet feces. 16S rRNA gene sequencing was performed on the collected samples to assess the relative contribution of various bacterial sources to the piglet gut microbiota. Overall, this study provides insight into the origin of gut bacteria and new information about the development of the gut microbiota in piglets.

## Methods

### Animal Experiments

Samples were collected from April to May 2017. Throughout the study, all animals were housed under similar conditions on a commercial farm in Guangdong Province, China. The pens in the unit were furnished with a polypropylene plastic slatted floor with 3.91 m^2^ space per sow. Thirty healthy Large-White/Landrace pregnant multiparous sows with similar expected delivery dates were selected and intramuscularly injected with cloprostenol (0.2 mg per sow) on day 113 of gestation to ensure synchronous delivery. Candidate sows that differed in delivery time by more than 3 h were excluded. In total, 20 sows with litters of 10 or 11 piglets were used in this study. Upon delivery, 60 infant pigs (3 piglets per litter per sow) were cohoused with sows by litter and ear notched for individual identification, following standard husbandry practices for swine. Sows were given *ad libitum* access to feed at 8:30 am and 2:30 pm and received water freely throughout the day, while suckling piglets had free access to water after 7 days. The infant piglets were allowed to nurse freely until weaning at 21 days of age without creep feed and did not consume sow feed. On weaning day, sows were removed from the piglets, and the piglets remained in the nursing pens for one week until the end of the experiment at day 28 to avoid the stress caused by environmental changes. None of the studied sows or piglets required antibiotics during the sampling period.

### Sample Collection

**FIGURE 1:**
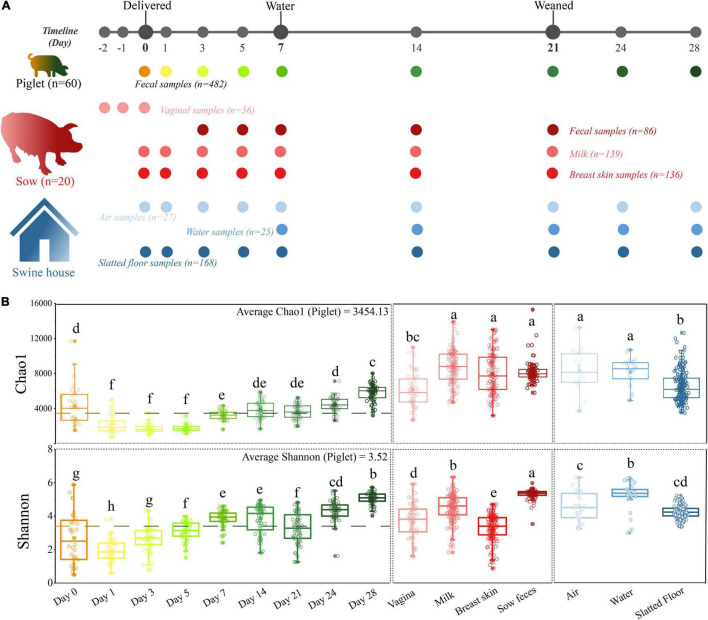
A longitudinal exploration of microbiomes between both sow-piglet pairs and housing environment. **(A)** Overall workflow of sample collection from piglets, environment, and sows at each sampling time point. All sows (*n* = 20) and their piglets (*n* = 60, three piglets per litter) were co-raised in the same environment. **(B)** Microbial alpha-diversity in different sample types. Different letters indicate significant differences (one-way ANOVA, *P* < 0.05) in the mean values.

A total of 482 piglet fecal samples, 86 sow fecal samples, 139 milk samples, 56 vaginal swabs and 136 breast skin swabs from sows, 25 water samples, 27 indoor airborne samples, and 168 slatted floor samples were collected at days 0, 1, 3, 5, 7, 14, 21, 23, and 28 (Figure 1A and Supplementary Table 1). Fecal samples were collected rectally from each piglet using a sterile cotton swab (Huachenyang, Shenzhen, China) premoistened with sterile phosphate-buffered saline (PBS), and then the swab head was placed in a 5 mL sterile screw-top collection tube (Corning, NY, United States). Sow feces were collected on days 3, 5, 7, 14, and 21 because they did not defecate on days 0 and 1 after delivery. Vaginal swabs were collected on days −1 and −2 (prepartum) and day 0 (before delivery). The vulva was cleaned with water and wiped with 75% ethyl alcohol to remove contaminating bacteria. The vaginal introitus was swabbed in a circular motion 5 times, and then the swab head was placed in a 5 mL sterile screw-top collection tube. Bacterial samples from the skin surface were collected by swabbing the anterior, middle, and posterior parts of the sow’s breast skin surface for approximately 30 s in a back-and-forth motion with swabs premoistened with sterile PBS, and then the swab head was placed in a 5 mL sterile screw-top collection tube. After the breast skin swabs were collected, the nipple and surrounding area of the sow were cleaned with soap and sterile water and then cotton soaked with 75% ethyl alcohol to minimize contamination with skin bacteria. Milk samples (approximately 15 mL) were collected manually in a sterile tube after the first few drops (approximately 1 mL) were discarded.

Five water samples (approximately 1 L each) were collected from the water trough and placed into sterile containers at each sampling time. Indoor air samples were collected using an SKC Bio Sampler (SKC Inc., PA, United States), which was placed ∼50 cm above the floor. Three air sampling replicates were collected at 10:00, 14:00, and 18:00 h by drawing air through the impingers filled with sterile molecular-grade water for 1 h at a rate of 13 L per min. For each replicate, the air sample was collected five times simultaneously at five points indoors, and the resulting samples (10 mL each) were pooled (50 mL total volume). Slatted floor surface samples of each pen were collected from five sites. During this procedure, a swab premoistened with sterile PBS was rubbed back and forth several times at each sampling site, and then the swab head was placed in a 5 mL sterile screw-top collection tube. Samples were immediately placed in liquid nitrogen after collection, transferred to the laboratory within 24 h, and stored at −80°C until DNA extraction.

### DNA Extraction, Library Preparation, and Sequencing

DNA was extracted using a QIAamp DNA Stool Mini Kit (Qiagen, Hilden, Germany) or PowerWater DNA Isolation kit (MoBio, Hilden, Germany). For samples collected via swabbing (feces, skin, slatted floor, and vagina), the swab head was placed in a bead tube containing 0.7 mL of sterile PBS. The tube was centrifuged at 15,000 × *g* for 10 min, and the pellets were suspended in 1.4 mL of ASL buffer from the DNA isolation kit. Zirconium glass beads (400 mg; diameter, 0.1 mm; BioSpec, Bartlesville, OK, United States) were added to the suspension that was shaken twice vigorously using a FastPrep-24 Instrument (MP Bio, Santa Ana, CA, United States) at a speed of 6.0 m/s for 2 × 45 s. The mixture was then incubated at 95°C for 5 min to maximize bacterial DNA extraction. All remaining steps followed the manufacturer’s protocol of the DNA stool mini kit. For the milk samples, a total of 2 mL of milk sample was thawed on ice and centrifuged at 15,000 × *g* for 10 min to separate the fat and cells from the whey. Then, DNA was extracted from pellets as described above.

For DNA extraction with a PowerWater DNA isolation kit, the water samples and impinger liquid from air samples were vacuum filtered onto sterile 0.2 μM polycarbonate filters (Sigma, St. Louis, MO, United States) and transferred to 0.7 mm garnet bead tubes containing 1 mL PW1 solution. The mixture was shaken vigorously using a FastPrep-24 Instrument, and the remaining steps followed the manufacturer’s protocol.

Extracted DNA was used as a template for PCR using barcoded primers to amplify the V4 region of the 16S rRNA gene. The V4 region of the 16S rRNA gene was amplified using universal primers 515F/806R (Parada et al., 2016), where PCRs were carried out in triplicate using 10 μM primer 515F and 806R, 1 × GoTaq Green Master Mix (Promega), 1 mM MgCl_2_, and 3 μL of DNA template or nuclease-free water as a negative control. The amplification conditions included an initial denaturation of 3 min at 94°C, followed by 35 cycles of 94°C for 45 s, 50°C for 60 s, and 72°C for 90 s, and a final extension for 10 min at 72°C. PCR products were pooled at equimolar concentrations and purified using a QIAquick PCR purification kit, and 2 × 250 bp read sequencing was performed on the Illumina HiSeq 2500 platform. Raw sequencing data in this study have been deposited in the European Nucleotide Archive database with the accession number PRJEB28241.

### The 16S rRNA Gene Sequence and Statistical Analysis

The raw reads of 16S rRNA gene sequencing were demultiplexed and quality-filtered using the Quantitative Insights Into Microbial Ecology (QIIME) program (v 1.9.1). Reads were trimmed and removed based on quality scores < 25 and lengths > 225 bp, respectively (Caporaso et al., 2010). Chimeras and error sequences were removed using QIIME software (v1.9.1) and the remaining reads were clustered into operational taxonomic units (OTUs) with a 97% similarity cutoff (Edgar, 2010). Taxonomy was assigned in QIIME using the default classifier workflow based on an Illumina-curated version of the Greengenes database. The OTU table was normalized by rarefaction at 7,500 sequences per sample prior to downstream analyses (MG-RAST v3; Meyer et al., 2008). The Chao1 diversity index was estimated with the “fossil” package and the Shannon diversity index was calculated with the “vegan” package in R (v3.0.3). Differences in the alpha diversity metrics between sample types were assessed using a one-way analysis of variance (ANOVA) with Kruskal-Wallis *post hoc* test and Benjamini-Hochberg FDR correction. To represent the distance between samples, non-metric multidimensional scaling (NMDS) based on Bray-Curtis dissimilarity was calculated using MG-RAST v3, in which the non-parametric permutational multivariate analysis of variance (PERMANOVA) was used to test differences among groups. A similarity percentages (SIMPER) analysis was conducted in MG-RAST v3 to measure the differences in bacterial communities between groups and to identify which taxa were primarily responsible for the differences. An unweighted pair group method with arithmetic averages (UPGMA) clustering analysis was performed at the genus level with relative abundance data using MG-RAST v3 to compare microbiota compositions (Suzuki and Shimodaira, 2006). Cooccurrence patterns of genera were constructed in the network interface by Spearman’s rank correlations based on bacterial abundance. A valid cooccurrence event was based on strong (Spearman’s *r*_*s*_ < −0.7 or *r*_*s*_ > 0.7) and significant (*P* < 0.01) correlations between genera. PICRUSt (1.1.3) was used to predict metagenome function by 16S rRNA marker gene sequences against the Greengenes database v13.5 (Langille et al., 2013). The OTU table used for PICRUSt was generated by picking closed reference OTUs with QIIME. The other procedures including OTU normalization followed the default parameters in PICRUSt. Linear discriminant analysis (LDA) effect size (LEfSe) was used to identify microbiota and functional genes showing differential abundance between groups (α = 0.05 and with an LDA score > 3.0). BugBase was used to predict the microbial phenotype with default parameters (Ward et al., 2017).

### Analysis of the Sources of Bacterial Taxa in Piglet Guts Using SourceTracker

The relative contribution of maternal and environmental substrates to the assembly of bacteria in the piglet gut was analyzed with SourceTracker (V.1.0) with default parameters (Knights et al., 2011). OTUs present in less than 1% of samples were first filtered the piglet feces at different ages were set as the “sink” and the samples from sows (feces, milk, breast skin, and vagina) and surrounding delivery environment (air, water, and slatted floor) were identified as the “source” regardless of sampling time. The results were aggregated into three categories, vagina, milk, and other (breast skin, sow feces, water, air, slatted floor, and unknown), and visualized as ternary plots with the R package ggtern.

### Modeling the Maturation Process of Gut Microbiota Using the Random Forest Algorithm

Random Forest models were used to regress relative abundances of OTUs in the time-series profile of the microbiota of piglets against their chronologic age, using default parameters of the R implementation of the algorithm (R package “randomForest,” ntree = 10,000, using default mtry of *p*/3 where *p* is the number input 97%-identity OTUs (features; Gao et al., 2017). The Random Forest machine-learning algorithm was used to determine a ranked list of all bacterial taxa in the order of age-discriminatory importance. The “rfcv” function was applied over 100 iterations to estimate the minimal number of top-ranking age-discriminatory taxa required for prediction. A sparse model with 30 top OTUs was selected based on 10-fold cross-validation. A smoothing spline function was fit between microbiota age and chronologic age of the piglet (at the time of fecal sample collection) in the validation sets to which the sparse model was applied.

## Results

### Characteristics of the Study Samples

In our study, 1,119 samples were collected and used for downstream analysis including piglet feces (*n* = 482), sow feces (*n* = 86), milk (*n* = 139), vaginal (*n* = 56), breast skin (*n* = 136), water (*n* = 25), air (*n* = 27) and floors (*n* = 168; Figure 1A and Supplementary Table 1). The microbiome of all samples was analyzed by 16S rRNA gene sequencing, yielding 74,032,942 high-quality sequences after quality control, with an average of 66,160 ± 391 sequences per sample (ranging from 7,746 to 92,011). The overall number of OTUs detected was 40,533 based on ≥ 97% nt identity. Rarefaction curves based on the Chao1 and Shannon diversity index of all samples nearly reached a plateau, indicating that the sampling depth was sufficient to characterize the bacterial communities (Supplementary Figure 1).

### The Similarity of the Microbial Community Structure Between Sample Types

Alpha diversity analysis revealed that the richness and diversity of the microbial communities were distinct at different sources. The Chao1 index of the piglet microbiome was significantly lower than all environmental and maternal microbiomes except for the vaginal microbiome, and the Shannon diversity index of the sow fecal microbiome was significantly higher than that of other samples (*P* < 0.05, Figure 1B). In addition, we observed higher alpha diversity values in the piglet fecal microbiome at the first time point (day 0) compared to day 1. However, these values then increased over the duration of the study.

**FIGURE 2:**
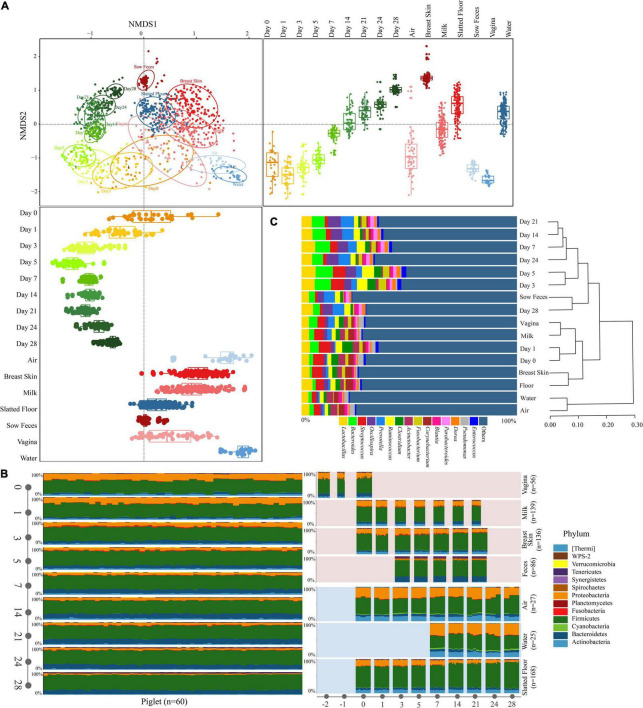
Characteristics of the microbiota in all types of samples. **(A)** NMDS plot of Bray-Curtis dissimilarity between different types of samples. Bray-Curtis dissimilarity was calculated using the abundance of OTUs. Groups from different types were significantly different by a PERMANOVA analysis on Bray-Curtis dissimilarity (*p* = 0.001, stress = 0.16). Box-and-whisker plots shown along each NMDS axis represent the median and interquartile range with whiskers determined by Tukey’s method, indicating the distribution of samples along the given axis. **(B)** Relative abundance of the phyla in different types of samples. **(C)** The relative abundance of the bacterial taxonomy in different types of samples (≥ 1.0% of the total sequences) and hierarchical clustering using the UPGMA method performed on a Bray-Curtis dissimilarity matrix at the genus level. Reads representing < 1.0% of the total were pooled and labeled “others.”

**FIGURE 3:**
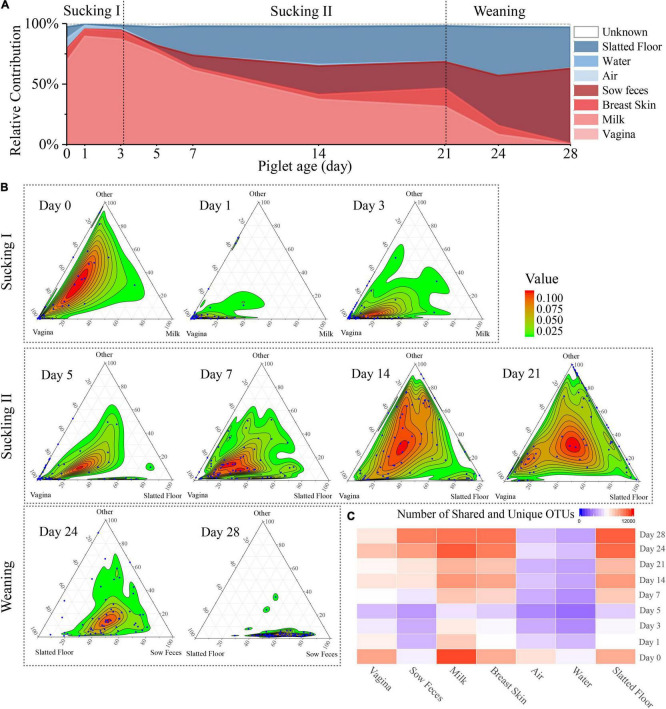
Source of piglet fecal microbiota at nine sampling times. **(A)** Proportion of microbiota from piglet feces at different days that estimated the origin from different sow sources (milk, feces, breast skin, vagina) and environmental source (air, water, slatted floor). **(B)** Three-axis ternary plots indicating the proportion of OTUs within a piglet’s fecal sample (each point) that is predicted to originate from sow or environmental samples (indicated by the triangle vertices). Each blue point represents a piglet fecal sample, and its position indicates the predicated relative contribution from the sow’s vagina, milk, or other sow samples (breast skin, sow feces) or environmental samples (air, water, slatted floor, or unknown source). The two most important microbial sources for each sampling time were selected as vertices, and the other sources were labeled as “others.” Points closer to the vertices indicate that a greater proportion of the sample’s OTUs is predicted to originate from the microbiota of the indicated sow sample or environmental sample. **(C)** Heatmap of the number of shared OTUs between piglet fecal samples by sampling time and other sample sources.

The NMDS ordination based on Bray-Curtis dissimilarity showed distinct clusters between the sample types, and the early piglet microbiome did not consistently resemble one specific sow or environmental sample (stress = 0.16, Figure 2A). For example, the early piglet fecal samples (at days 0 and 1) clustered with the sow vaginal samples, while they gradually shifted toward sow fecal samples as the piglets aged. PERMANOVA showed that sample type significantly affected the bacterial community structure (*R*^2^ = 0.82, *P* = 0.001). The fecal microbiomes in the piglets were relatively divergent from each other and had high intersubject variability, particularly on days 0, 1, and 3, compared with those of the sows (Figure 2A and Supplementary Figure 2). SIMPER analysis on the microbial community dissimilarity further confirmed the NMDS and PERMANOVA results (Supplementary Figure 3). SIMPER analysis of the sow and environmental microbiota compared with the piglet fecal microbiota indicated that the piglet fecal microbiota on day 0 was more similar to the vaginal, milk, and breast skin microbiota than to the other microbiota groups. This high similarity between the piglet feces (day 0) and the vagina, milk, and breast skin was attributed to Proteobacteria (range from 30.3 to 41.1%, Supplementary Tables 2–4). The similarity between the piglet fecal and sow vaginal microbiota increased within the first three days after birth and then gradually decreased, but the similarity between the piglet fecal and the sow fecal microbiota gradually increased as the piglets aged. On day 28, the piglet fecal microbiota was more similar to the sow fecal microbiota (the value of average Bray-Curtis dissimilarity was 0.093) than to other samples, which was attributed to the dominance of Firmicutes (36.3%, Supplementary Table 5 and Figure 3). The UPGMA clustering analysis at the genus level showed that the fecal microbiota on days 0 and 1 clustered with sow vaginal samples and the fecal microbiota on day 28 clustered with sow fecal samples (Figure 2C). Firmicutes, Proteobacteria, Bacteroidetes, and Actinobacteria were the four most relatively abundant phyla in all samples except in sow feces and accounted for 93.6–95.9% of the 16S rRNA gene sequences in all sample types (Figure 2B and Supplementary Table 6). Firmicutes, Bacteroidetes, Tenericutes, and Spirochaetes were the most relatively abundant phyla and accounted for 94.0% of the sow fecal microbiota. At the genus level, 23, 20, 25, 21, 22, 26, 22, and 26 predominant bacterial taxa (average relative abundance of > 1%) were identified in piglet feces (65.6% of the total sequences), sow feces (78.9%), milk (52.5%), breast skin (53.7%), vagina (55.4%), air (50.9%), slatted floor (55.9%) and water (55.6%) samples, respectively (Figure 2C).

### SourceTracker Analysis Highlights the Contribution of Sow and Environmental Sources to the Piglets

SourceTracker, a Bayesian probability tool [29], was used to predict the relative contributions of the sow and delivery environment microbiota to the piglet fecal microbiota. The results revealed that the vaginal microbiota contributed the most to the meconium (day 0) microbiota compared with other sources, followed by the slatted floor (9.6%), milk (9.4%), and air (8.5%; Figure 3A). The relative contribution of the vaginal microbiota to the piglet fecal microbiota increased in the first 3 days from 69.0 to 89.3% and then gradually decreased to 0.28% on day 28. Interestingly, the relative contribution from sow feces gradually increased after day 5 and finally reached the highest on day 28 (62.1%). However, the relative contribution of bacteria from sow milk was increased only on days 0 (9.4%) and 21 (15.0%). Apart from the vertical transmission of the sow microbiota, the neonatal piglets were also exposed to a wide variety of environmental microbiota. The slatted floor contributed 18.1% of the bacterial communities on day 0, rapidly decreased in contribution to 4.0% on day 3, and gradually increased in contribution to 34.1% on day 28, indicating that the slatted floor was the primary environmental source of bacterial communities in piglets, especially 5 days after birth, while air and water contributed less to the colonization of piglet bacterial communities than the slatted floor.

Ternary plots were used to reflect the contribution of various bacterial sources more intuitively to each fecal microbiome of piglets on different days. As shown in the plot, the piglet fecal samples were more closely related to the sow vaginal sample on the first 7 days (Figure 3B). The piglet fecal samples diverged in their distributions among the vertices in the ternary plot at days 14 and 21, indicating that the bacterial sources during these times were more complex. On day 24, almost all fecal samples were uniformly distributed between the slatted floor and sow fecal samples, and most of the piglet fecal samples were close to the sow fecal samples in the plot at day 28. Analysis of the OTU co-occurrence patterns showed a hierarchy among the sample sources that were shared with piglet feces (Figure 3C). This result indicates that more OTUs in piglet feces were shared by the milk, breast skin, and slatted floor samples than by the water and air samples. As the piglets aged, the similarity and the number of OTUs shared between the piglet and sow fecal microbiota increased, which might be related to the increase in diversity (Drell et al., 2017). Overall, the relative contribution of various sources of bacteria to the microbial composition of the piglet gut gradually changed as the piglets aged, and the main source of microbes in the fecal microbiota of the piglets was the vagina of the sow within 3 days after birth, which was gradually replaced by the sow feces and the slatted floor.

### Maturation of the Piglet Fecal Microbiota

**FIGURE 4:**
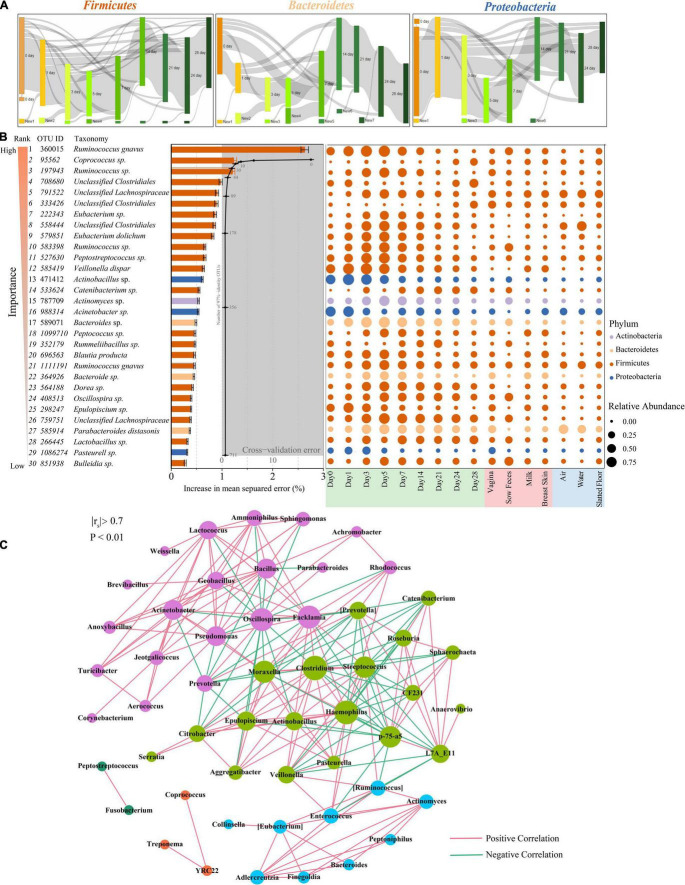
Bacterial taxonomic biomarkers for defining gut-microbiota maturation in piglet feces during the first 4 weeks of life. **(A)** OTUs that are shared by at least 10% of the population within each time point are tracked using Sankey plots in Firmicutes, Bacteroidetes, and Proteobacteria. The heights of the rectangles indicate the relative number of OTUs, and each time point has a distinct color. The lines represent the transfer of OTUs between time points and are colored by the first day of appearance. **(B)** Thirty age-discriminatory bacterial taxa were identified by applying Random Forests regression of their relative abundances in fecal samples against chronologic ages in 60 piglets. The color of rank from light to dark represents the importance from low to high. Shown are OTUs ranked in order of their importance to the accuracy of the model. The insert shows a 10-fold cross-validation error as a function of the number of the input OTUs regressed against the age of piglets in the training set. Heatmap of mean relative abundances of the 30 age-predictive bacterial taxa plotted against the chronologic age of piglet used to train the Random Forest model. **(C)** Network of co-occurring genera within piglet feces. The nodes represent the genera, and the size of each node is proportional to the degree (the number of connections). The edges stand for strong and significantly positive (red) or negative (green) correlations between genera. The nodes are colored based on modularity structure.

Tracking individual OTUs within the three dominant phyla revealed distinct temporal dynamics (Figure 4A). Many of the Firmicutes OTUs displayed dynamic volatility, with 16.4% disappearing between days 0 and 1; 77.8% of those that disappeared eventually reappeared after day 14 (Figure 4A, left panel). A smaller proportion of the Bacteroidetes and Proteobacteria OTUs also showed dynamic changes. The greatest number of Bacteroidetes OTUs disappeared at days 0 and 1 (49.3%) but reappeared after day 7 (Figure 4A, middle panel). The greatest number of Proteobacteria OTUs disappeared from days 3 to 5 (35.1%) but reappeared after day 24 (Figure 4A, right panel). We used BugBase to further predict phenotypes in the piglet fecal microbiomes (Ward et al., 2017). BugBase predicted that the fecal microbiome of the piglets had a higher proportion of facultative anaerobic bacteria than obligate aerobic and obligate anaerobic bacteria on days 0 and 1 (Supplementary Figure 4). The proportion of anaerobic and facultative anaerobic bacteria showed a contrasting trend, in which the proportion of anaerobic bacteria gradually increased from 22.3 to 68.0% during the first 4 weeks postpartum, while the proportion of facultative anaerobic bacteria gradually decreased from 55.75 to 11.8%. The proportion of aerobic bacteria in the piglet fecal microbiomes was only 15.2% at day 0, and this proportion decreased over the first five days postpartum before recovering over time (Supplementary Figure 4).

The relative abundances of OTUs were regressed against the chronologic age of each piglet using the Random Forest machine-learning algorithm to probe the age-dependent development of the piglet fecal microbiota. The regression explained 98.4% of the variance related to chronologic age. The top ranking age-discriminatory taxa were selected according to their variable importance measures using 10-fold cross-validation. Thus, the top 30 age-discriminatory taxa were identified and used for the subsequent construction of the microbiota-based model for discriminating the degree of microbiota maturity, as the inclusion of any taxa beyond these top taxa produced only minimal improvement in model performance (Figure 4B). This model consisted of 21 genera that distinguished the maturity of the gut microbiota during the 28 days of the experiment. Although the natural development of the gut microbiota exhibited a smooth curve that gradually increased, the curve did not reach a plateau until day 28 (Supplementary Figure 5), indicating that the gut microbiota had not reached maturity by the end of this study. The significantly changed taxa across sampling times mainly belonged to the *Lachnospiraceae* and *Erysipelotrichaceae* families (Figure 4B).

To explore bacterial interactions within piglet feces and environment samples, we used network analysis based on strong (Spearman’s *r*_*s*_ < −0.7 or *r*_*s*_ > 0.7) and significant (*P* < 0.01) correlations of genera. In this network, it was assumed that co-occurring genera interacted with each other in either a positive or negative manner. The piglet feces network consisted of 53 nodes (genera) and 211 edges (relations) with an average degree (the mean number of connections per node) of 3.98 (Figure 4C). According to the modularity algorithm, the piglet feces genera were partitioned into five modularity structures, where major age-discriminatory taxa such as *Actinomyces* and *Bacteroides* were part of the same sub-community and had positive correlations. In addition, *Actinobacillus*, *Epulopiscium*, and *Pasteurella* were also major age-discriminatory taxa, which were part of the same sub-community and positively correlated. Most of the piglet fecal age-discriminatory taxa were also identified in the network of other sow and environmental samples (Supplementary Figure 6).

### Diversification of the Microbial Community Function

**FIGURE 5:**
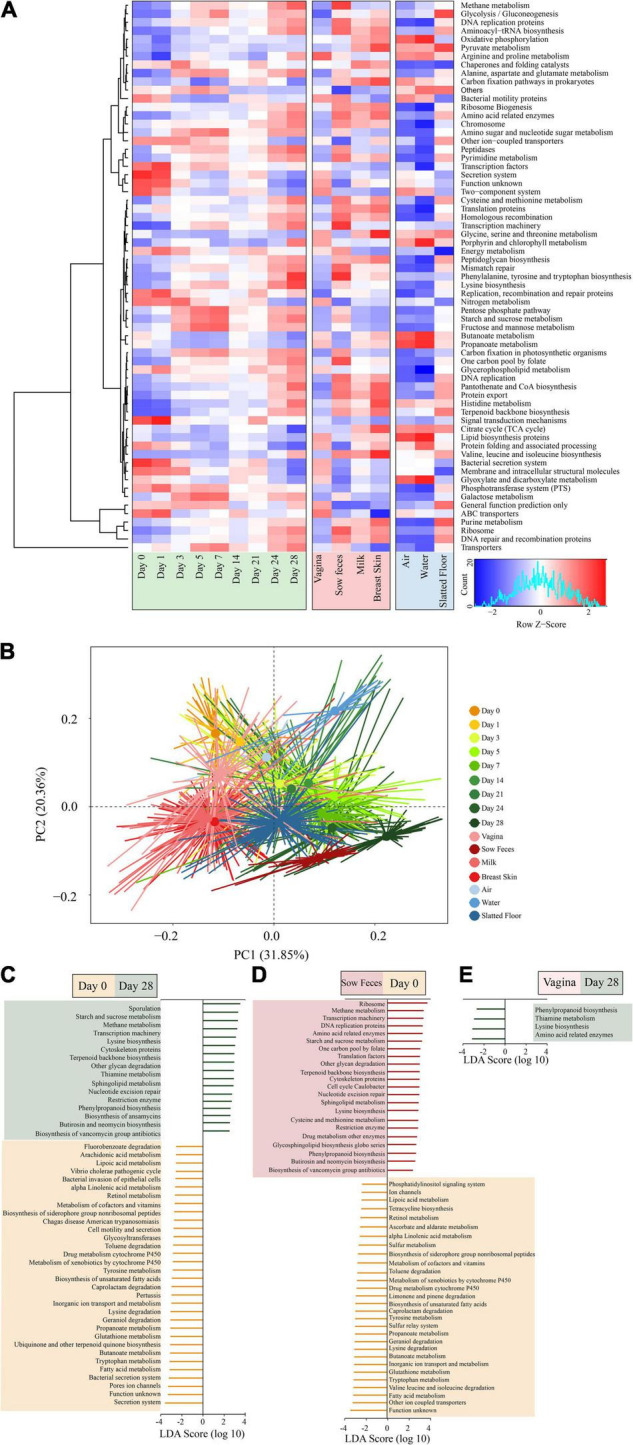
Metagenomic functional predictions for microbiota in different samples. **(A)** Heatmap showing distinct microbial gene (according to KEGG pathway analysis at the third level; >0.5% of the total sequences) profiles in the samples from piglet feces, sow (stool, vagina, breast skin, and milk), and environment (air, water, and slatted floor). **(B)** Principal coordinate analysis (PCA) of microbial functional diversity across the time points using the relative abundances of functional pathways. *P* < 0.001 by permutational analysis of variation (PERMANOVA). Results from LEfSe analysis based on the PICRUSt data set (third level), which was conducted to identify pathways that differentiated functional pathways between **(C)** the piglet feces at different time points (left), **(D)** piglet feces at the 0 day and sow feces (middle), and **(E)** the piglet feces at the 28 days and vaginal samples (right). Modules with linear discriminant analysis (LDA) score >3.0 are plotted.

We next sought to examine how the microbial metabolic and functional pathways of the early piglet fecal metagenome may have changed over time. The majority of the predicted functional genes of the piglet fecal microbiota were associated with transporters (6.90%), ABC transporters (3.58%), and DNA repair and recombination proteins (2.66%; Figure 5A and Supplementary Table 7). The relative abundance of transporters was also the highest in the other samples (Supplementary Table 7). Principal coordinates analysis (PCoA) with Bray-Curtis dissimilarity showed that the predicted functional profiles of the piglet fecal microbiota clustered more closely to the vaginal microbiota of sows at days 0 and 1, while they were more similar to the sow fecal microbiota at days 24 and 28 (Figure 5B). The LEfSe analysis revealed that 63 differentially abundant bacterial functions were present across the piglet sampling times (Supplementary Figure 7).

The piglet fecal microbiota at day 0 was predicted to be enriched for several microbial pathways, including secretion systems, pore ion channels, bacterial secretion systems, fatty acid metabolism, tryptophan metabolism, and butanoate metabolism. In comparison, the piglet fecal microbiota at day 28 was significantly enriched for pathways related to sporulation, metabolism, and biosynthesis, including starch and sucrose metabolism, methane metabolism, lysine biosynthesis, and terpenoid backbone biosynthesis (Figure 5C). There were no significant differences in the metabolic pathways of functional genes in the microbiota among piglet feces at day 0, sow vaginal samples, and the piglet and sow feces at day 28 according to the LEfSe analysis. However, 50 differentially abundant bacterial functions were observed between the piglet and sow feces at day 0 (Figure 5D). Metabolic functions, including fatty acids, tryptophan, glutathione, butanoate, valine, leucine, isoleucine, lysine, geraniol, and caprolactam degradation, were predicted to be overrepresented in the piglet fecal microbiota. In contrast, ribosome, methane metabolism, transcription machinery, DNA replication proteins, and amino acid-related enzymes were underrepresented in the sow feces. The relative abundance of phenylpropanoid biosynthesis, thiamine metabolism, lysine biosynthesis, and amino acid-related enzymes in the piglet fecal microbiota at day 28 compared to the vaginal microbiota of sows (Figure 5E).

## Discussion

The gut microbiota of mammals rapidly develops after parturition through microbial exposure. In our study, we investigated the sources of early colonization and development of the piglet gut microbiota from day 0 to 28 using a 16S rRNA gene sequencing approach. We found that the maternal vaginal microbiota was the primary source of the piglet gut microbiota during the first 3 days after birth, but this main source was gradually replaced by the sow fecal and slatted floor microbiota. These processes reflected the powerful selective forces of the host or adaptations of the different sources of the microbiota.

The high alpha diversity in the piglet gut microbiota at birth reflects the diverse microbiota, mainly from the sow’s vagina, which is consistent with previous studies (Bäckhed et al., 2015; Wampach et al., 2017; Ferretti et al., 2018). Vaginally delivered infants are first exposed to the maternal vaginal microbiota, which results in neonatal gut colonization by vaginal microbes (Biasucci et al., 2010; Dominguez-Bello et al., 2010). In our study, the relative contribution of the vaginal microbiota rapidly decreased after 3 days and was gradually replaced by the sow fecal and environmental microbiomes (Figure 3A), which was consistent with the decrease in the initial alpha diversity and intrasubject diversity of the gut microbial diversity in the piglets. The uterus of mammals is anaerobic, but the infant gut has a small amount of oxygen at birth and gradually becomes anaerobic (Houghteling and Walker, 2015; Rodríguez et al., 2015). These results suggest that the gut microbial colonization of piglets is a process of niche selection, which was confirmed by the initial decrease in the diversity of microbiota (Figure 1B) and the increased proportion of facultative anaerobic bacteria (Supplementary Figure 4).

The relative contribution of the vaginal microbiota gradually decreased over time, indicating that microbiota from the vagina was not suited to colonize in the gut of the piglet over the long term. Although these bacteria are present only transiently in the piglet gut, they regulate the gut condition changes from aerobic to anaerobic, which is essential for the adult animal (Bäckhed et al., 2015; Houghteling and Walker, 2015). Most likely due to the shift in the gut environment to an anaerobic state, the relative abundance of strictly anaerobic bacteria increased over time. The relative contribution of the sow fecal microbiota gradually increased and became the main source of the piglet gut microbiota after day 21, suggesting that vertically transmitted microbes from the sow vagina to the piglet were more ecologically adaptable in the piglet gut compared with other sow-derived microbes before 21 days of age. This finding disagrees with a study of human infants in which the contribution of the maternal fecal microbiota to the anal microbiota of vaginally delivered infants gradually decreased within 30 days after delivery (Dominguezbello et al., 2016). The reason for the difference might be that the piglets were raised with their mothers for an extended period and had more frequent exposure to the sow fecal microbiota, also piglets are coprophagic and consume the sow’s feces. In addition to vertical transmission of the vaginal and gut microbiota, neonatal human babies are exposed to a myriad of other microorganisms from different body sites and other maternal sources (Shin et al., 2015; Dominguezbello et al., 2016; Tamburini et al., 2016). The relative contribution of the milk and breast skin microbiota to the piglets was lower than that of other sow sources of transmission, which was not consistent with previous reports on the sources of gut microbiota in breastfed infants (Pannaraj et al., 2017). This finding may be due to the previous study sequencing only communities from milk and areolar skin while ignoring other maternal or environmental sources, which may have influenced the results of the source-tracking estimates (Knights et al., 2011; Chen et al., 2018; Law et al., 2021).

In our study, the sum of the predicted percentages of all sources is greater than 97% for piglet gut bacteria and exceeded previous studies on gut microbial sources with human infants (Biasucci et al., 2010; Pannaraj et al., 2017), which may be due to the large sample size and the piglets and sows being co-raised under a relatively stable environment throughout the trial. Another reason might be the major differences in how piglets and human infants are raised. Other studies with pigs have also shown that milk has limited effects on the colonization of the piglet gut microbiota (Chen et al., 2018; Law et al., 2021). Overall, our results reinforce the importance of this vertical sow-to-piglet microbial transmission from multiple sources, and further studies are needed to elucidate the mechanism of microbial vertical transmission.

In addition to maternal sources, the environment surrounding the newborn is also a natural source of microbes that can colonize different body sites by frequent contact (Brooks et al., 2014, 2017). Our results showed that the slatted floor was the most important source of environmental microorganisms for the colonization of the piglet gut microbiota, and similar results were also observed in the studies of the human infant gut microbiota (Brooks et al., 2014; Shin et al., 2015). The activities of piglets and sows in the farrowing bed created a unique microbial environment in the slatted floor that differs from sows and environmental microbiomes (Figure 2A), indicating that the slatted floor is not simply a carrier for the vertical transmission of sow fecal microbes and might be also affected by other factors, such as feeding activities. In swine production, biological additives containing different microbes are commonly used to spray confinement swine buildings to reduce the emission of odor, dust, and bioaerosol and may also colonize slatted floors (Kim et al., 2006). Our results indicated that these biological additives need to be carefully selected before application, as this might affect the colonization of the piglet’s gut microbiota. However, further studies should be conducted to confirm this hypothesis. Previous studies using both culture-dependent and culture-independent methods showed that there are complex microbial communities in the air (Edmiston et al., 2005; Emerson et al., 2017). However, the contribution of air bacteria to the colonization of the piglet gut was very low in our study. The reason may be that the aerobic bacteria in the air did not adapt well to the anaerobic environment of the piglet gut.

The dramatic changes in the gut microbial communities and the function of such changes in piglets early in life were observed in our study, and the results were in agreement with those of a previous study (Frese et al., 2015; Wang et al., 2019; Jurburg and Bossers, 2021; Tang et al., 2021). With the growth of piglets, the gut microbiota increased in complexity, which is reflected in the increase in microbial richness and evenness. Our study is the first to explore the gut microbiota maturity of newborn piglets using a Random Forest regression model, although the results indicate that the intestinal microbiota of piglets did not reach maturation at day 28, indicating that more time is needed to reach a steady-state. Previous studies have shown that several factors can influence the maturity of gut microbiota, including probiotic and antibiotic feeding (Gao et al., 2017; Fang et al., 2018), health status (Xiong et al., 2017; He et al., 2019), and the time of solid food introduction (Tamburini et al., 2016; Chen et al., 2017). However, the potential factors affecting the gut microbiota maturity of newborn piglets still require further study. The capacity for carbohydrate digestion was enhanced, which is consistent with previous studies (Chu et al., 2017; Tang et al., 2021; Wu et al., 2021). Furthermore, the predicted significant increase in the relative abundances of KEGG pathways associated with amino acid metabolism, especially lysine biosynthesis, further support the enhanced capacity of protein digestion and absorption as the piglets aged. Considering that gut microbes utilize host nutrients for survival, the enhancement in gut bacterial capacity for degradation of carbohydrates and proteins may be the result of the increased intake of solid feed composed of more complex carbohydrates and proteins than those in sow milk.

### Study Limitations

Although we found that the contribution of sow and environmental microbial sources to the colonization of the gut microbiota in piglets changed with the growth of piglets, our work has several limitations. First, it is difficult to investigate the sources for the piglet gut microbiota at the species level and even the genus level due to the limitations of 16S rRNA sequencing. We attempted to use metagenomic sequencing in this study as well but due to the low biomass in some samples, such as early piglet feces, air, and water samples, we did not extract enough DNA to meet the requirements of metagenomic sequencing. More samples should be collected in further similar studies to conduct with metagenomic sequencing (Zhou et al., 2020; Chen et al., 2021).

Second, the gut microbiota composition could be influenced by the host metabolism. In our study, sows were treated with cloprostenol, which is commonly used in pig production systems and research experiments, to ensure synchronous delivery. This may have an impact on the gut microbiota of sows and their piglets. However, we used it for all sows in our study to ensure that the external effects on all animals used in this study are consistent. The details of these effects require further study.

Third, there were relatively few microbes in the air and water samples. Therefore, we need to use different DNA extract kits for the air and water samples. However, different kits might introduce bias for the microbial composition, which should be avoided as much as possible in future research.

## Conclusion

We comprehensively analyzed the relative contribution of sow and environmental microbial sources to the colonization of gut microbiota in piglets. Ordination and cluster analyses revealed that the gut microbiota of piglets is closely related to the vaginal microbiota at days 0 and 1, and gradually shifts toward the sow fecal samples over time. In addition, the proportion of anaerobic gut bacteria gradually increased, while that of facultative anaerobic bacteria gradually decreased. More importantly, the initial colonizers in piglets, especially within the first 3 days of life, largely originated from the sow vaginal microbiota and were gradually replaced with the sow fecal and slatted floor microorganisms as piglets aged according to SourceTracker analysis. These results indicate that the gut microbial succession of piglets is a process of niche selection. Furthermore, gut microbiota maturity revealed that the intestinal microbiota of piglets did not reach maturation at day 28, and more time is needed to reach a steady-state. These findings underscore the importance of sows and the rearing environment in the development of the piglet gut microbiome.

## Data Availability Statement

The datasets presented in this study can be found in online repositories. The names of the repository/repositories and accession number(s) can be found in the article/Supplementary Material.

## Ethics Statement

The animal study was reviewed and approved by Animal Experimental Committee of South China Agricultural University.

## Author Contributions

WC, JM, NL, JG, and JC conducted the sample collections. JM, WC, and YJ conducted bioinformatics analyses. WC and NL conducted the nucleic acid extractions. WC, JM, LD, and YJ were major contributors to the manuscript. All co-authors provided comments for the manuscript. JM, TL, and XL directed the overall research project. All authors read and approved the final manuscript.

## Conflict of Interest

The authors declare that the research was conducted in the absence of any commercial or financial relationships that could be construed as a potential conflict of interest.

## Publisher’s Note

All claims expressed in this article are solely those of the authors and do not necessarily represent those of their affiliated organizations, or those of the publisher, the editors and the reviewers. Any product that may be evaluated in this article, or claim that may be made by its manufacturer, is not guaranteed or endorsed by the publisher.

## Supplementary Material

Supplementary Figure 1 Rarefaction curve of samples from different origins. OTU-level rarefaction (observed richness) **(A–H)** and Shannon diversity index **(I–P)** curves of microbiota from different types of samples.

Supplementary Figure 2 Bray-Curtis dissimilarity distances (beta diversity) within or between sample types. Smaller values indicate a greater similarity within or between sample types.

Supplementary Figure 3 The average dissimilarity (%) between piglets’ feces and other groups by SIMPER analysis at the phylum level.

Supplementary Figure 4 BugBase was used to predict the proportion of aerobic, anaerobic, and facultative anaerobic bacteria within microbiomes of piglet fecal samples.

Supplementary Figure 5 Microbiota age predictions in the piglets used to train the 30 bacterial taxa model (each circle represents an individual piglet fecal sample).

Supplementary Figure 6 The network of co-occurring predominant genera within the different sample types: **(A)** vagina, **(B)** sow feces, **(C)** milk, **(D)** breast skin, **(E)** slatted floor, **(F)** water, and **(G)** air. The nodes represent the predominant genera, and the size of each node is proportional to the degree (the number of connections). The edges stand for strong (Spearman’s correlation coefficient *r*_*s*_ > | 0.7|) and significantly positive (red) or negative (green) correlations between core genera (*P* < 0.01). the nodes are colored based on module structure.

Supplementary Figure 7 Results from LEfSe analysis based on the PICRUSt data set (the third level), which was conducted to identify pathways that differentiated functional pathways of the piglet feces at different time points. Modules with linear discriminant analysis (LDA) score > 3.0 are plotted.

Supplementary Table 1 Overview of successfully sequenced samples (“−” = samples were not collected that day).

Supplementary Table 2 The similarity of percentages analysis of Bray–Curtis dissimilarity between piglet feces at day 0 and vagina.

Supplementary Table 3 The similarity of percentages analysis of Bray–Curtis dissimilarity between piglet feces at day 0 and milk.

Supplementary Table 4 The similarity of percentages analysis of Bray–Curtis dissimilarity between piglet feces at day 0 and breast skin.

Supplementary Table 5 The similarity of percentages analysis of Bray–Curtis dissimilarity between piglet feces on day 28 and sow feces.

Supplementary Table 6 Taxa composition of the piglet fecal, sow, and environmental microbiome. Average relative abundances and standard deviation (SD) of each identified.

Supplementary Table 7 The relative abundance of the predicted functions across different types of samples.
