# Supplementary figures and images for: Selective Maternal Seeding and Rearing Environment From Birth to Weaning Shape the Developing Piglet Gut Microbiome
Source: Front Microbiol. 2022 Apr 25;13:795101. doi: 10.3389/fmicb.2022.795101 (PMC9083071; doi:10.3389/fmicb.2022.795101)

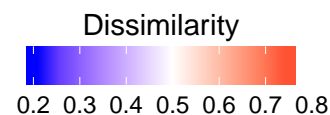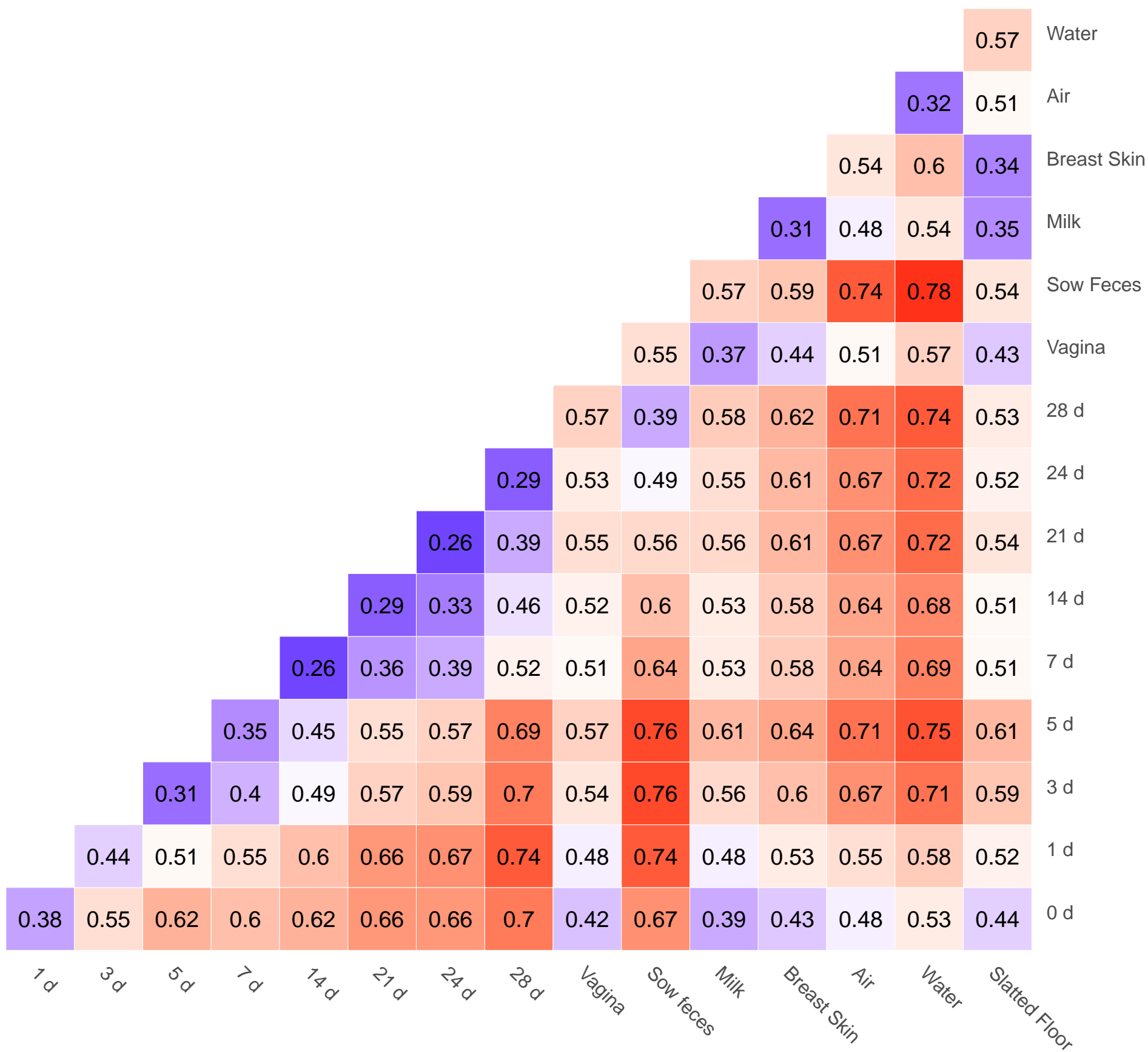

Supplement: Supplementary Figure 2 — Bray-Curtis dissimilarity distances (beta diversity) within or between sample types. Smaller values indicate a greater similarity within or between sample types. [file Image_2.PDF]

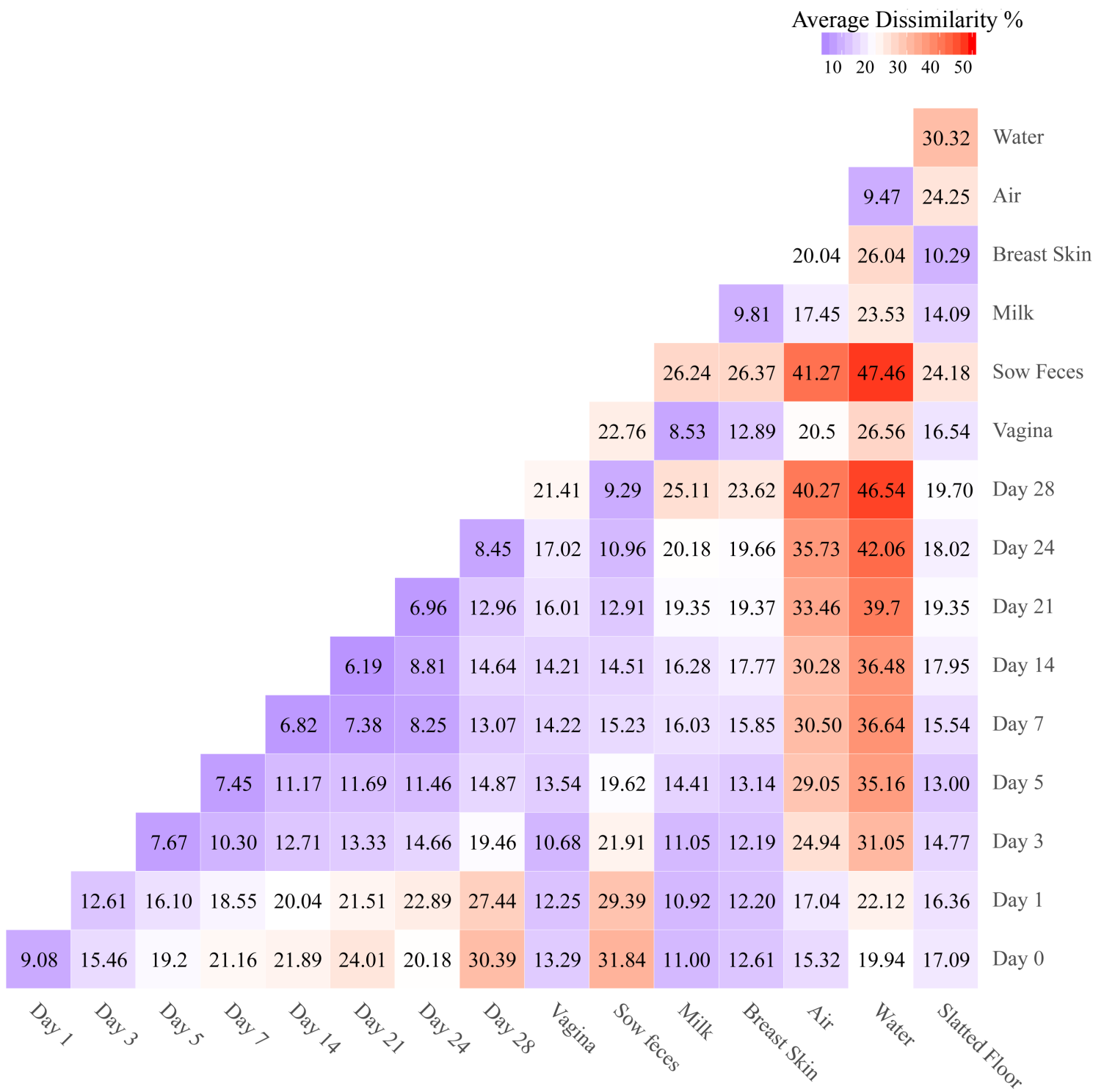

Supplement: Supplementary Figure 3 — The average dissimilarity (%) between piglets’ feces and other groups by SIMPER analysis at the phylum level. [file Image_3.PDF]

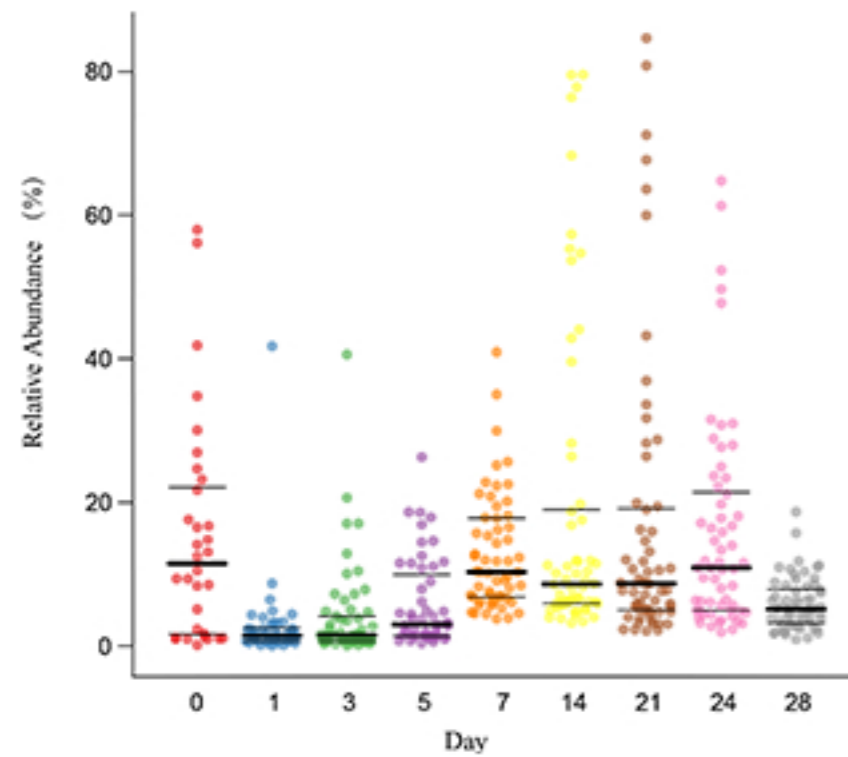

Aerobic bacteria

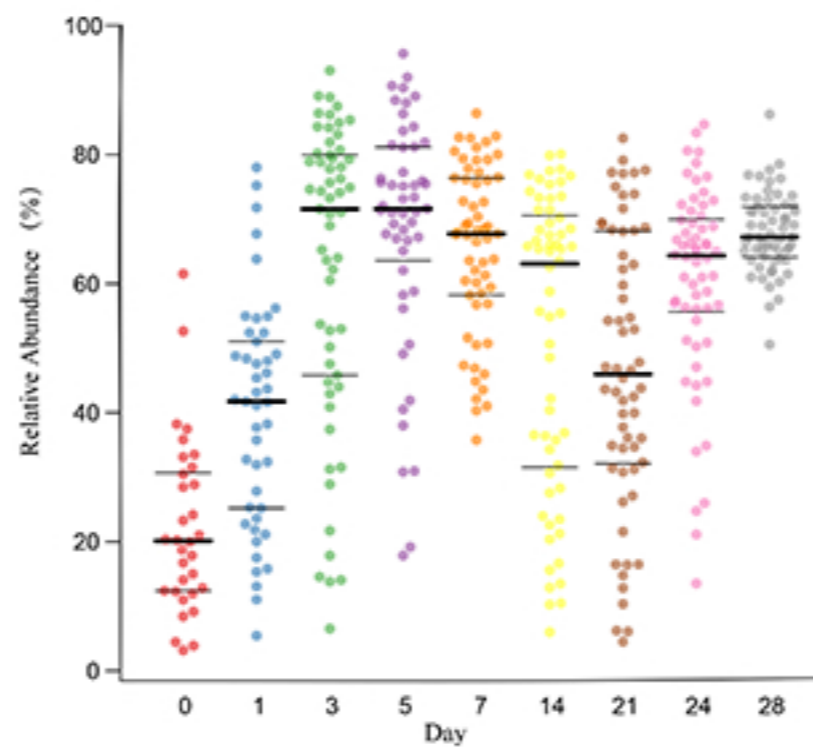

Anaerobic bacteria

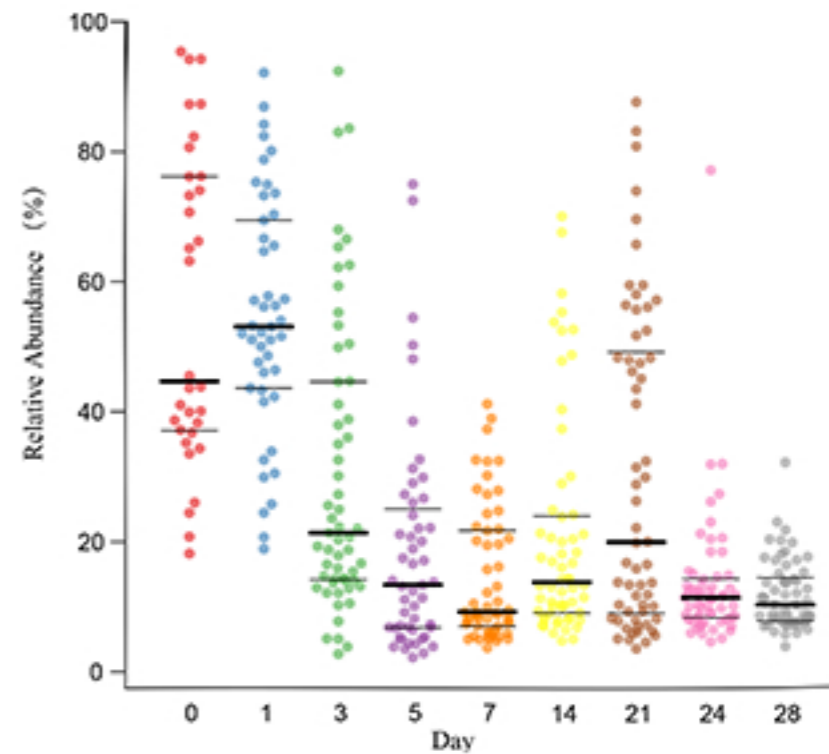

Facultatively anaerobic bacteria

Supplement: Supplementary Figure 4 — BugBase was used to predict the proportion of aerobic, anaerobic, and facultative anaerobic bacteria within microbiomes of piglet fecal samples. [file Image_4.PDF]

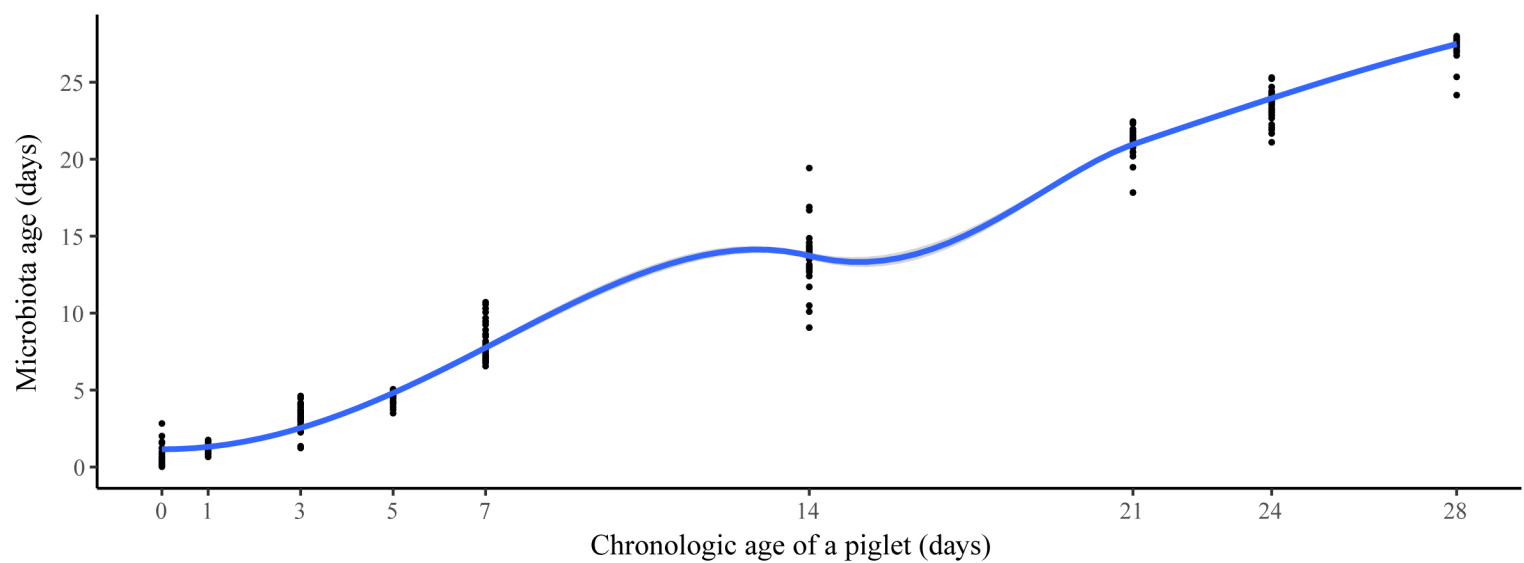

Supplement: Supplementary Figure 5 — Microbiota age predictions in the piglets used to train the 30 bacterial taxa model (each circle represents an individual piglet fecal sample). [file Image_5.PDF]

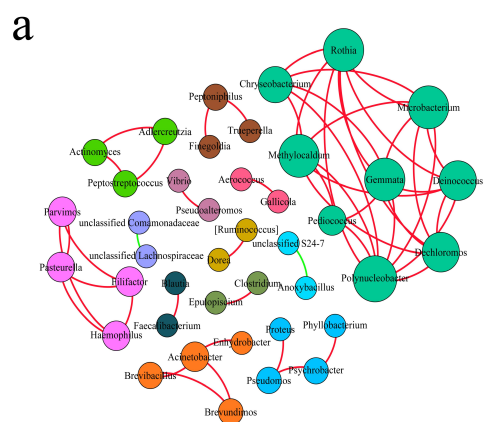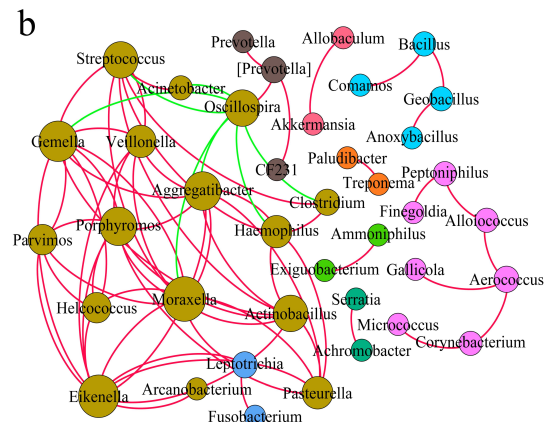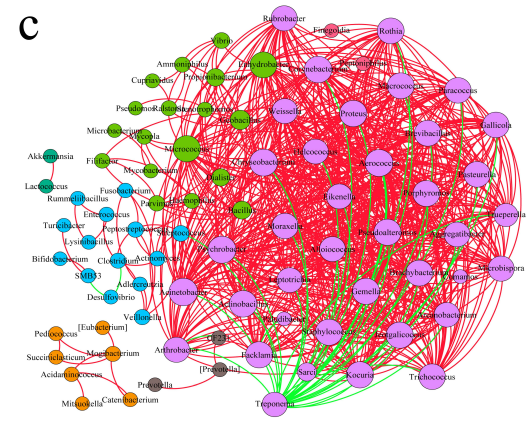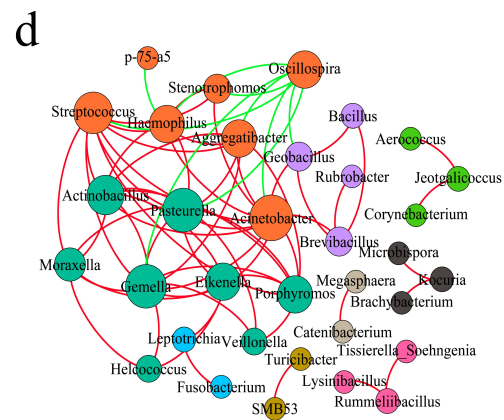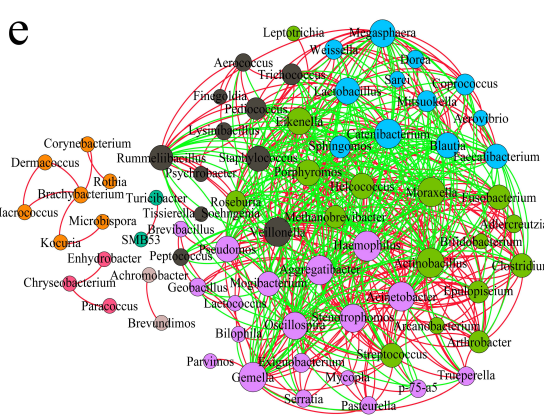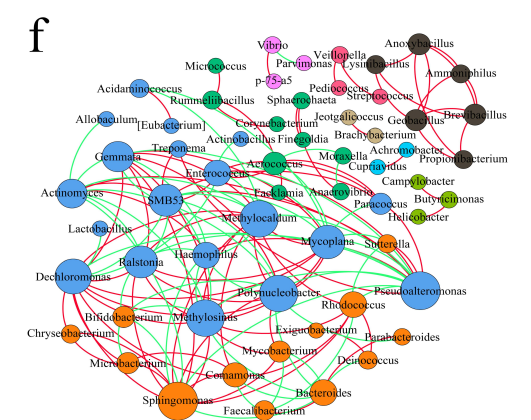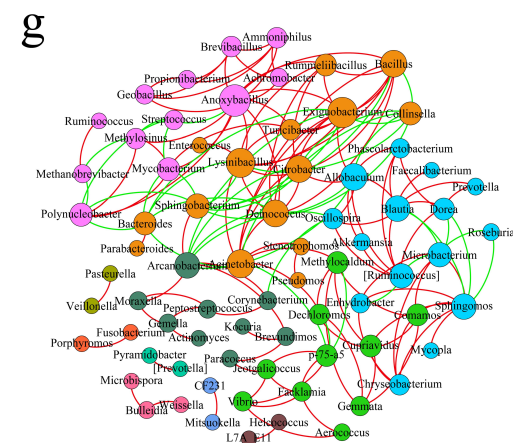
$$|\mathbf{r}_s| > 0.7 \quad \mathbf{P} < 0.01$$

———— Positive Correlation

———— Negative Correlation

Supplement: Supplementary Figure 6 — The network of co-occurring predominant genera within the different sample types: (A) vagina, (B) sow feces, (C) milk, (D) breast skin, (E) slatted floor, (F) water, and (G) air. The nodes represent the predominant genera, and the size of each node is proportional to the degree (the number of connections). The edges stand for strong (Spearman’s correlation coefficient rs > | 0.7|) and significantly positive (red) or negative (green) correlations between core genera (P < 0.01). the nodes are colored based on module structure. [file Image_6.PDF]

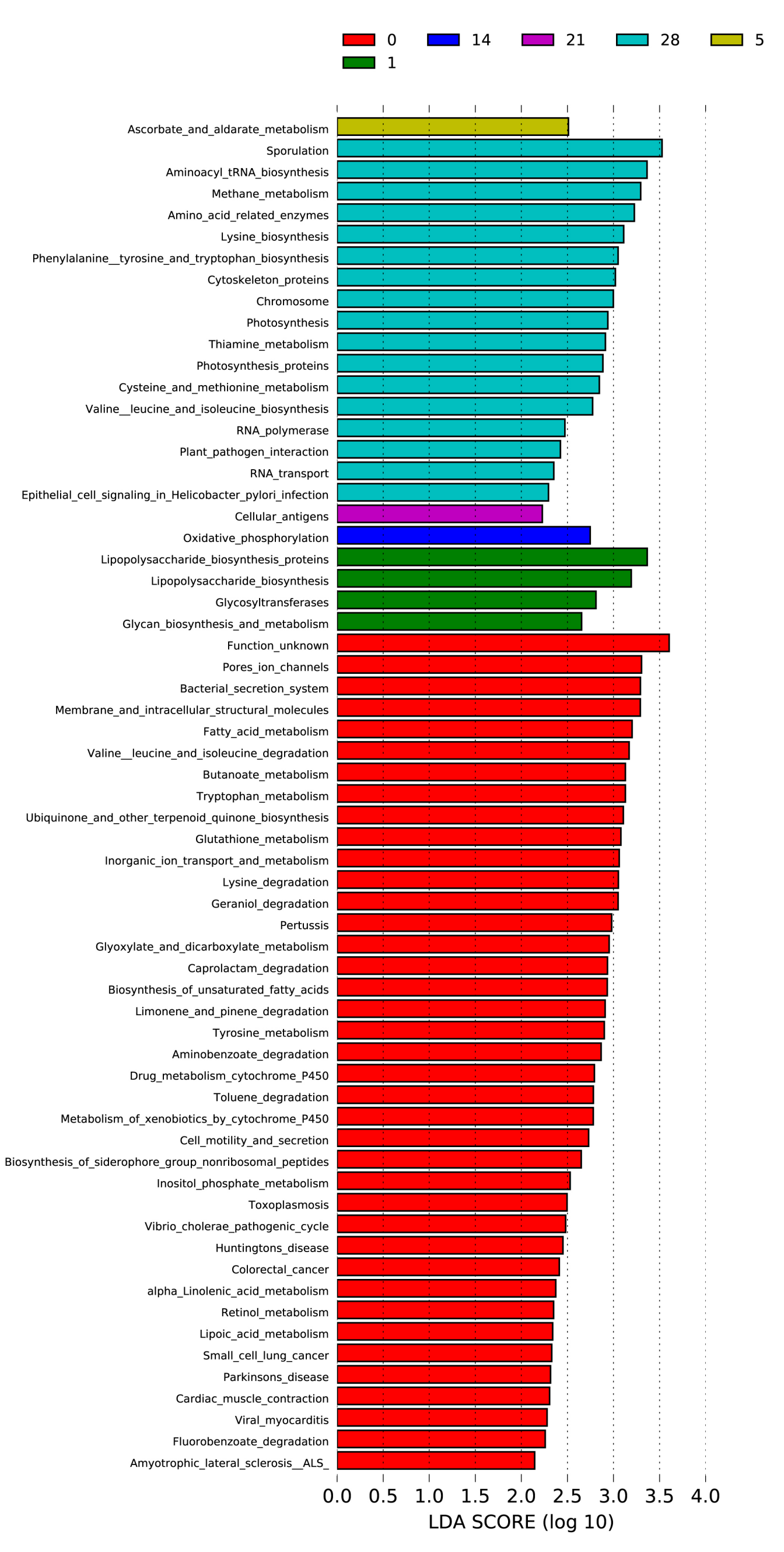

Supplement: Supplementary Figure 7 — Results from LEfSe analysis based on the PICRUSt data set (the third level), which was conducted to identify pathways that differentiated functional pathways of the piglet feces at different time points. Modules with linear discriminant analysis (LDA) score > 3.0 are plotted. [file Image_7.PDF]
