# Supplementary material for: Selective Maternal Seeding and Rearing Environment From Birth to Weaning Shape the Developing Piglet Gut Microbiome
Source: Front Microbiol. 2022 Apr 25;13:795101. doi: 10.3389/fmicb.2022.795101 (PMC9083071; doi:10.3389/fmicb.2022.795101)
Supplement: Supplementary Figure 1 — Rarefaction curve of samples from different origins. OTU-level rarefaction (observed richness) (A–H) and Shannon diversity index (I–P) curves of microbiota from different types of samples. [file Image_1.PDF]

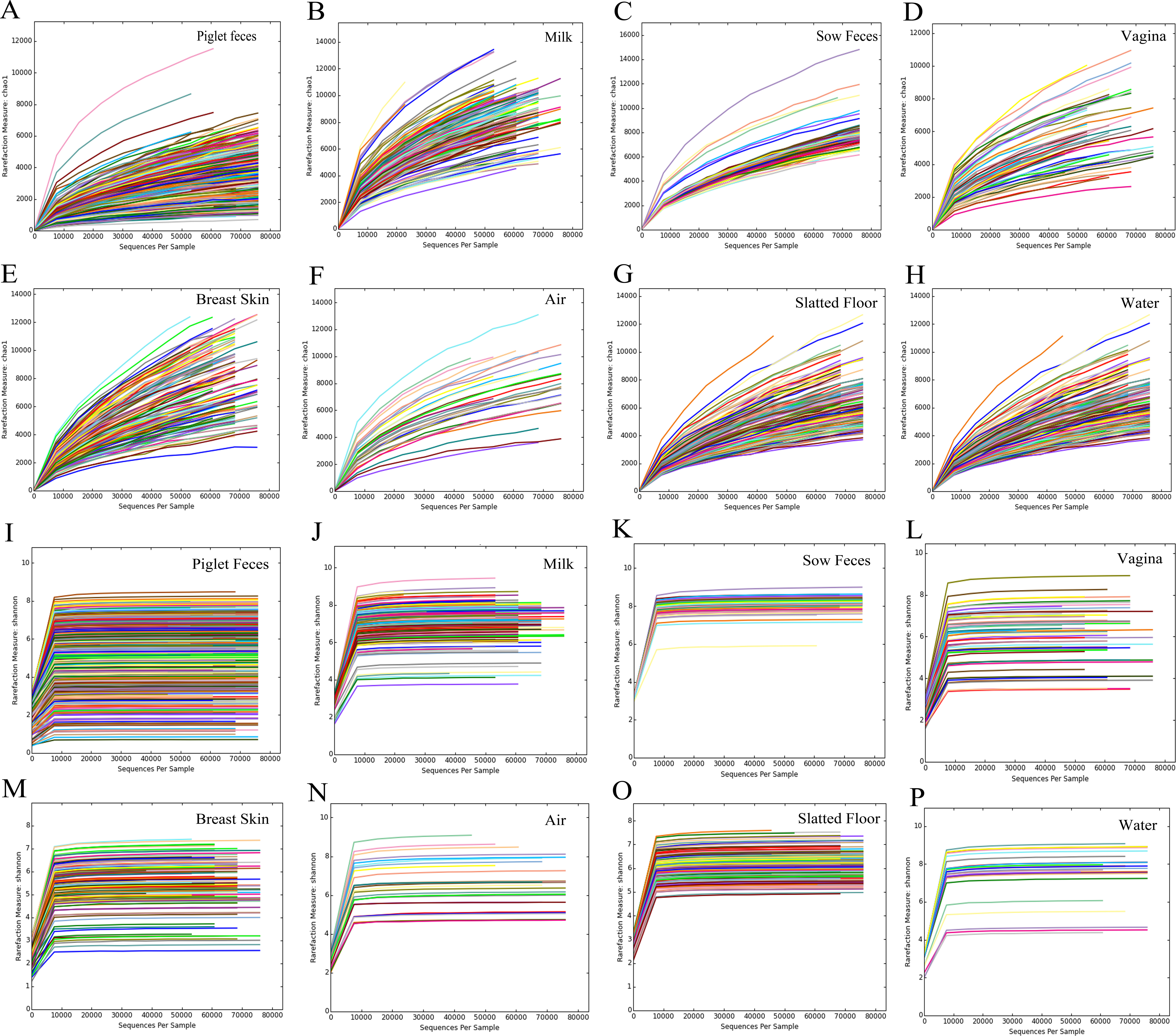

A
Piglet feces
Rarefaction Measure: chao1
Sequences Per Sample
B
Milk
Rarefaction Measure: chao1
Sequences Per Sample
C
Sow Feces
Rarefaction Measure: chao1
Sequences Per Sample
D
Vagina
Rarefaction Measure: chao1
Sequences Per Sample
E
Breast Skin
Rarefaction Measure: chao1
Sequences Per Sample
F
Air
Rarefaction Measure: chao1
Sequences Per Sample
G
Slatted Floor
Rarefaction Measure: chao1
Sequences Per Sample
H
Water
Rarefaction Measure: chao1
Sequences Per Sample
I
Piglet Feces
Rarefaction Measure: shannon
Sequences Per Sample
J
Milk
Rarefaction Measure: shannon
Sequences Per Sample
K
Sow Feces
Rarefaction Measure: shannon
Sequences Per Sample
L
Vagina
Rarefaction Measure: shannon
Sequences Per Sample
M
Breast Skin
Rarefaction Measure: shannon
Sequences Per Sample
N
Air
Rarefaction Measure: shannon
Sequences Per Sample
O
Slatted Floor
Rarefaction Measure: shannon
Sequences Per Sample
P
Water
Rarefaction Measure: shannon
Sequences Per Sample
